# ExceS-A: an exon-centric split aligner

**DOI:** 10.1515/jib-2021-0040

**Published:** 2022-03-07

**Authors:** Franziska Reinhardt, Peter F. Stadler

**Affiliations:** Bioinformatics Group, Institute of Computer Science, Interdisciplinary Center of Bioinformatics, Leipzig University, Härtelstraße 16-18, D-04107 Leipzig, Germany; Max-Planck-Institute for Mathematics in the Sciences, Inselstraße 22, D-04103 Leipzig, Germany; Institute of Theoretical Chemistry, University of Vienna, Währingerstraße 17, A-1090 Wien, Austria; Facultad de Ciencias, Universidad National de Colombia, Sede Bogotá, Colombia; Santa Fe Institute, 1399 Hyde Park Rd., Santa Fe, NM 87501, USA

**Keywords:** AUTS2, ExonMatchSolver, paralogs, split aligner

## Abstract

Spliced alignments are a key step in the construction of high-quality homology-based annotations of protein sequences. The exon/intron structure, which is computed as part of spliced alignment procedures, often conveys important information for the distinguishing paralogous members of gene families. Here we present an exon-centric pipeline for spliced alignment that is intended in particular for applications that involve exon-by-exon comparisons of coding sequences. We show that the simple, blat-based approach has advantages over established tools in particular for genes with very large introns and applications to fragmented genome assemblies.

## 1 Introduction

Accurate multiple sequence alignments (MSA) are needed to study the evolution of protein families and to trace the changes in shape, function, dynamics, structure, and folding throughout the history of particular gene. In the case of gene families with multiple paralogs, this in particular also requires an unambiguous assignment of paralog groups, a task that is sometimes difficult based on the mRNA or protein sequences alone. Differences in the exon/intron structure are often useful to disambiguate paralogs. In contrast, there are orthologs in many eukaryotic species that preserve the exon-intron structure. These “splicing orthologs” [1] are abundant. Already a decade ago, about a third of the human transcripts could be associated with a splicing ortholog in mouse [2]. Since both transcript coverage and completeness of gene annotations have improved dramatically over the last decade, the levels of splicing orthology are presumably much higher.

Workflows such as ExonMatchSolver [3] attempt to address these issues by identifying homologs together with their exon/intron structure, and to use this information to improve the assignment of paralogs. Such approaches can be used to construct paralog-specific exon-wise sequence alignments. Converted to HMMs, these can be used to improve gene annotations and orthology assignments for multi-gene families.

A key step in such workflows is the spliced alignment of a query, usually a protein sequence, against a genomic region. Splice alignment is a well-studied task for which a diverse set of tools is already available. The task is of particular importance for RNA-seq read mapping [4]. Nowadays, most mapping tools natively support the detection of split reads. Nevertheless, spliced alignment of protein queries to genomic DNA has remained an important issue e.g. in genome annotation.

ProSplign [5] utilizes a global alignment algorithm extending the classical Needleman–Wunsch dynamic programming approach with high penalties for frame shifts, and low extension cost for introns. Exonerate [6] employs bounded sparse dynamic programming, providing a fast approximation to the exact solution of the alignment problem. In genome-wide applications, these approaches need to be embedded in workflows that identify candidate regions before deploying the dynamic programming algorithms. Neither tool explicitly considers the existence of (plausible) splice sites, start codons, or stop codons to assess the completeness of the predicted exons. Scipio [7], on the other hand, uses blat [8] to find candidate hits in the target genome and employs a series of hit-refinement steps and tries to locate very short exons that are missing the initial blat search before filtering the hits and assembling the final result. Scipio also searches for splice sites and terminal exons. A practical inconvenience of all three tools is that they only report the predicted protein sequence but not its subdivision into exons, which have to be re-computed from the genomic coordinates. More importantly, no scoring of the splice sites is provided, hence alternative splice sites resulting in similar predicted sequences on the target genomes cannot be compared without extensive processing. More recent spliced alignment tools such as SplicedFamAlign [9] focus on the CDS-to-gene alignment problem and therefore take nucleic acid sequences rather than protein sequences as input, and hence cannot be readily used for our purposes.

Despite the high quality of the results of the available tools for its intended applications, we found that their output requires a level of post processing and manual curation that is prohibitive at least for comprehensive studies of large gene families or complete proteomes. We therefore developed a spliced alignment pipeline based on blat [8] with the specific aim of facilitating the construction of exon-specific HMMs. The ExceS-A pipeline combines ideas of several well-established tools and is designed to simplify the necessary post-processing and to support a fully automatic processing of large-scale data.

## 2 ExceS-A: exon-centric spliced alignments

In contrast to conventional spliced alignment tools, ExceS-A starts from a query protein sequence annotated by exon boundaries. The pipeline starts with an initial blat search of the individual query exons against a user-defined subset of the target genome. For our main applications, both the subset of the target genome and the exon/intron boundaries of the query protein are computed with ExonMatchSolver [3]. The target is not necessarily a single, contiguous stretch of genomic sequence but may consist of (parts of) multiple contigs. ExceS-A assumes, however, that these target sequence contains only a single homolog of the query protein. This initial blat comparison uses the parameter maxIntron = 20,000 and supports the identification of intron insertions relative to the exon/intron structure of the query. This step is intended to narrow down the region(s) in the target on which the spliced alignment has to be performed and to identify approximate locations of the exons. The resulting blat hits are then filtered. Exons lacking >12 amino acids at the beginning or >10 at the end of the alignment when compared to the query are removed from the initial set to exclude spurious sequences.

The second step is the identification of exons that were not recovered in the initial blat search. To this end, temporary target sequences are constructed by extracting the regions between exons on the target genome that are retained after the initial blat search. If first or last exons are missing, the corresponding temporary target sequences extends to begin or end, resp., of the target sequence provided as initial input. Missing exons are used as queries for a blast [10] search against the target sequence in which the exon is expected according to synteny. We use blast for this step because it is more sensitive than blat. The increased computational effort for blast is compensated by the much shorter target sequence. Only blast hits with a score >50.0 bits are considered.

If two exons map in close proximity on the target we check whether or not there is an intervening intron. If two consecutive exons are separated by less than 5 nt, they are assumed to be a single exon. This cutoff is very conservative, given that “ultra-short” spliceosomally-processed introns are extremely rare [11–13]. In the output nevertheless reports two consecutive exons and only annotates the merge since separate sequences and alignments are required for the intended application scenarios.

After identifying the distinct exons, their boundaries are refined. To this end, we search for start codons (ATG) in the first exon, stop codons (TAG, TAA, and TGA) in the last exon and splice junctions near the exon ends. The canonical pattern GT...AG is the most abundant, appearing e.g. in 99.24% of mammalian introns [14], with GC...AG being the most common alternative (0.69% in mammals). The signature pattern of splicing by the minor spliceosome, AT...AC, see e.g. [15], and all other non-canonical splice junctions are exceedingly rare. The current implementation of ExceS-A therefore only considers G[T|C]...AG. This search is carried out in a radius of 35 nt around the initial estimate for the exon end, or to the 5′ or 3′ end, resp., of the target sequence that contains the candidate exon.

The candidate splice sites are then scored with MaxEntScan [16], which evaluates the short sequence motifs around the 5′-site (donor) or 3′-site (acceptor). Reliable donor and acceptor candidates have positive scores and [17] showed that a cutoff >3 already leads to highly reliable predictions on conserved splice sites. We also accept very poor scores, >−10 , as possible sites if no better alternative can be found.

**Figure 1:**
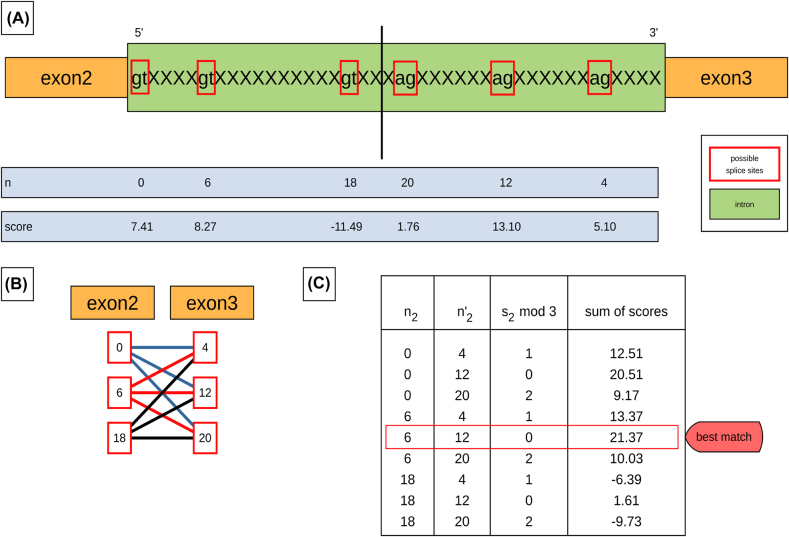
Determining the combined MaxEntScan score and the relative reading frame for each pair putative donor and acceptor site. (A) Assuming exon2 and exon3 are neighboring exons, *n*_2_ is the number of sequence positions between the last nucleotide of the first amino acid of exon2 and the candidate 5′ splice site. Correspondingly $n_{2}^{\prime }$ is the number of sequence positions between the putative 3′ splice site and the first nucleotide of the first amino acid of exon3. (B) Every possible splice site of one exon is matched to the corresponding possible splice sites of the neighboring exon. (C) The sum $s_{2}=n_{2}+n_{2}^{\prime }$ is tabulated for all possible splice junctions. The pair of donor and acceptor candidates preserved the reading frame if and only if *s*_2_ mod 3 = 0, i.e., if *s* is divisible by 3. The match with the highest score is assumed to be the correct splice junction of the two neighboring exons.

The splice junction is then determined as follows: for each pair of putative donor and acceptor site the combined MaxEntScan score and the relative reading frame is determined. Denote by *n*_*i*_ the number of nucleotides between the last nucleotide of the last amino acid predicted by blat for the *i*th exon and the candidate for the 5′ splice junction. Correspondingly, $n_{i}^{\prime }$ denotes the number of nucleotides between the candidate for the 3′ splice junction and the first nucleotide of the first amino acid predicted by blat for the (*i* + 1)st exon. Note that the numbers *n*_*i*_ and $n_{i}^{\prime }$ can by negative. This is the case if the candidate for the 5′ splice site is located by the predicted end of the exon *i*, and if the 3′ splice site is locate after the predicted begin of the exon *i* + 1. Writing $s_{i}≔n_{i}+n_{i}^{\prime }$ , we observe that the predicted combination of 3′ and 5′ splice sites preserves the reading frame if and only if *s*_*i*_ is divisible by 3, i.e., if *s*_*i*_ mod 3 = 0 (Figure 1).

If no candidate with a splice site score >−10 can be found, the search radius is extended once by 18 nt and the best admissible combination of splice junctions is computed for the extended region. If no splice site with a score >−10 can be found, the corresponding exon is rejected and a warning is issued that situation may require manual curation. For exons predicted on the same target sequence, a minimum intervening intron length of 21 nt is assumed; otherwise the intervening sequence is treated as a readthrough.

**Figure 2:**
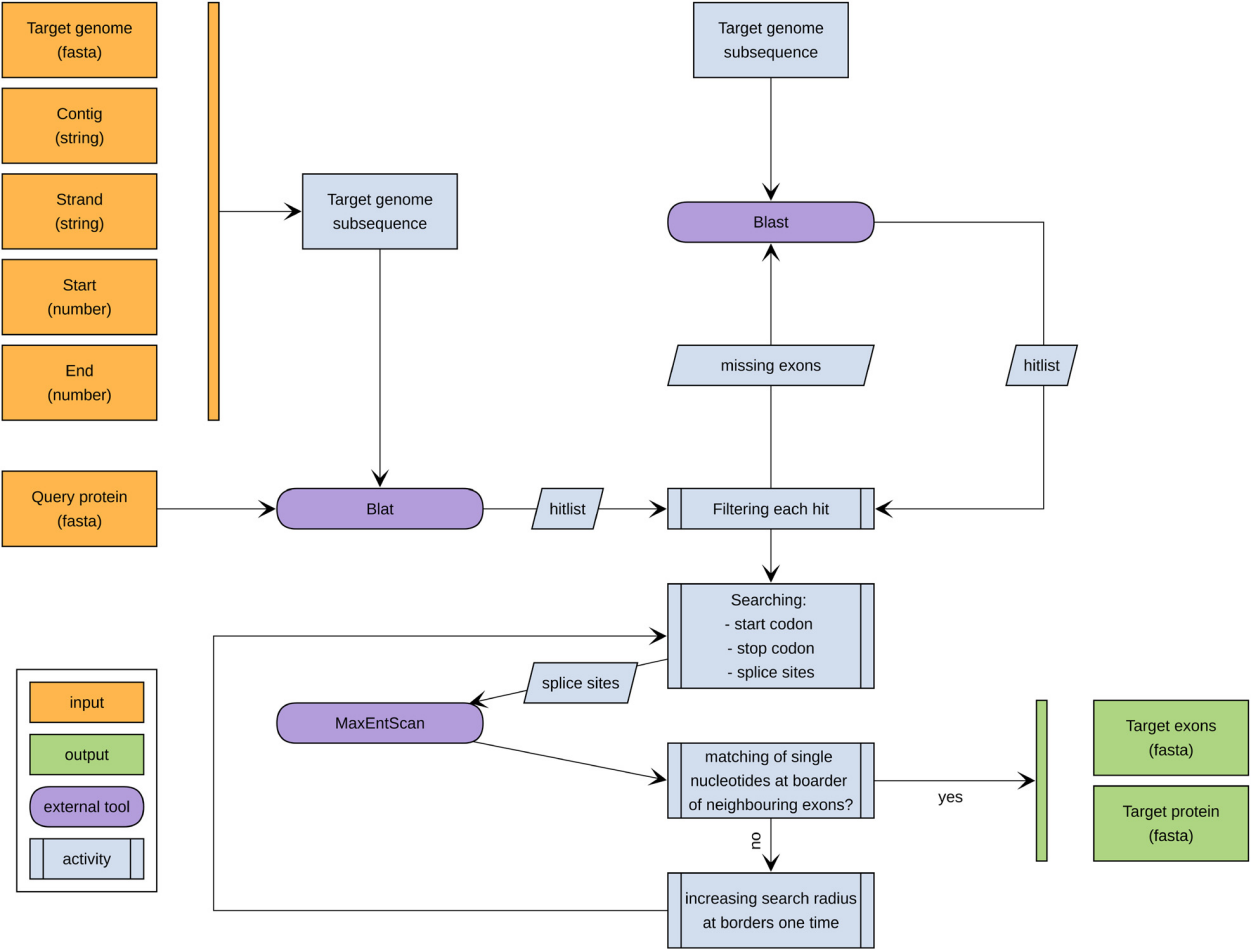
Overview of the ExceS-A workflow.

In its output, ExceS-A reports both the complete predicted protein sequence on the target DNA and the sequences of the individual exons. In case of the exon sequences, amino-acids whose codons are disrupted by splice junctions are not show. These amino acids are of course included in the sequence reported for the complete protein, however. The workflow of ExceS-A is summarized in Figure 2. The tool is implemented in perl. It requires BLAT, Blast, MaxEntScan and BioPerl.

## 3 Application: automatic generation of exon-level HMMs for protein homology search

The ExonMatchSolver pipeline uses spliced alignments twice. First, the chained hits of the initial homology search with tblastn are subdivided into exons on single contig or limited-size genomic region. The latter is important for genes with very long introns for performance reasons. In this step, the query protein is compared to a single DNA region to identify one or more chained exons. In the new version of the ExonMatchSolver pipeline, ExceS-A replaces Exonerate and ProSplign for this task, incurring a substantial performance gain. The core algorithm of ExonMatchSolver then solves the Paralog-to-Contig Assignment problem [3] for a collection of protein fragments in an incompletely assembled genome, thus producing for each paralogous protein a collection of DNA sequences that harbor its exons. The pipeline now employs ExceS-A to extract the final intron-exon boundaries, making it obsolete to use Scipio for this purpose. The workflow of the current version of ExonMatchSolver, indicating the usage of ExceS-A, is shown in the Supplemental Figure S1.

**Figure 3:**
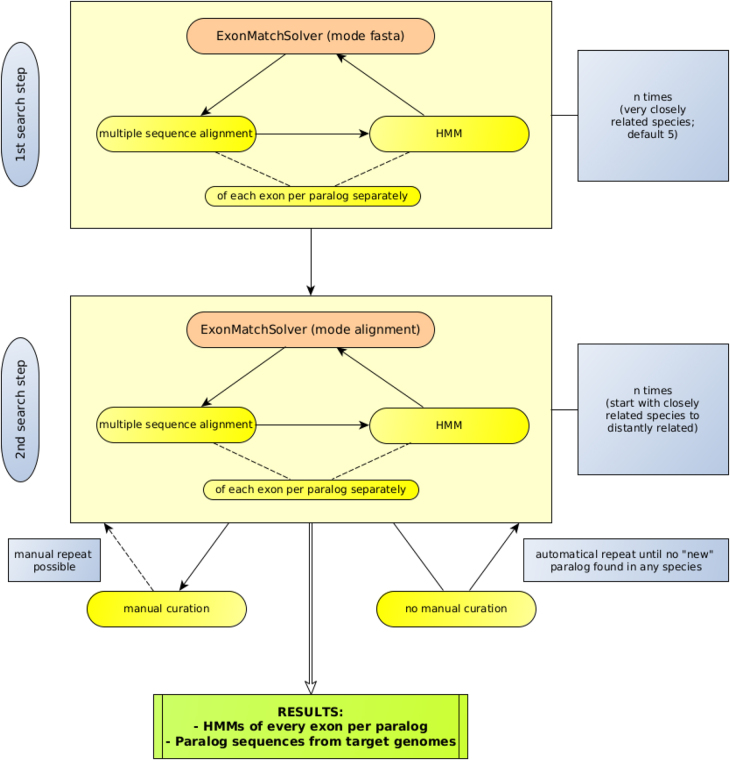
Automation. The automation of the ExonMatchSolver (EMS) search in a group of species is divided in two steps. First search with the mode fasta of the EMS to create the hidden Markov models (HMMs) for the second search step which uses the alignment mode of the EMS. The second search step is repeated with all species still lacking at least one of the sought paralogs until no new paralog sequence is found in any species.

In order to generate exon-centric alignments of a single paralog group of a protein family, we embedded ExonMatchSolver into a workflow that explored a set of phylogenetically related genomes in the following manner, (Figure 3). In the initial step we use the “fasta mode” of ExonMatchSolver to find homologs of the known paralogous proteins from a single query species to retrieve the orthologs for each them in a few (default *n* = 5) very closely related species. ExonMatchSolver produces, separately for each paralog group, a multiple sequence alignment (MSA) and a corresponding HMM for each exon. In the second step, these HMMs are used as input for the “HMM mode” of ExonMatchSolver, which now searches for matches of the individual exon HMMs and combines these matches into (co-)orthologs for each paralogous queries. This results in exon-wise MSAs augmented by the newly identified exon sequences. Revised HMMs are derived from these MSAs. These can then be used for a new round of homology search. This search can also include the previously searched genomes, so that the HMMs trained on phylogenetically more diverse sequences can be used to identify additional paralogs that may not have been present in the initial query set. The iteration terminates when not further homologs are found. The pipeline also offers the possibility to export the MSAs for manual curation and re-start the iterative process with MSAs and HMMs modified externally by the user.

**Figure 4:**
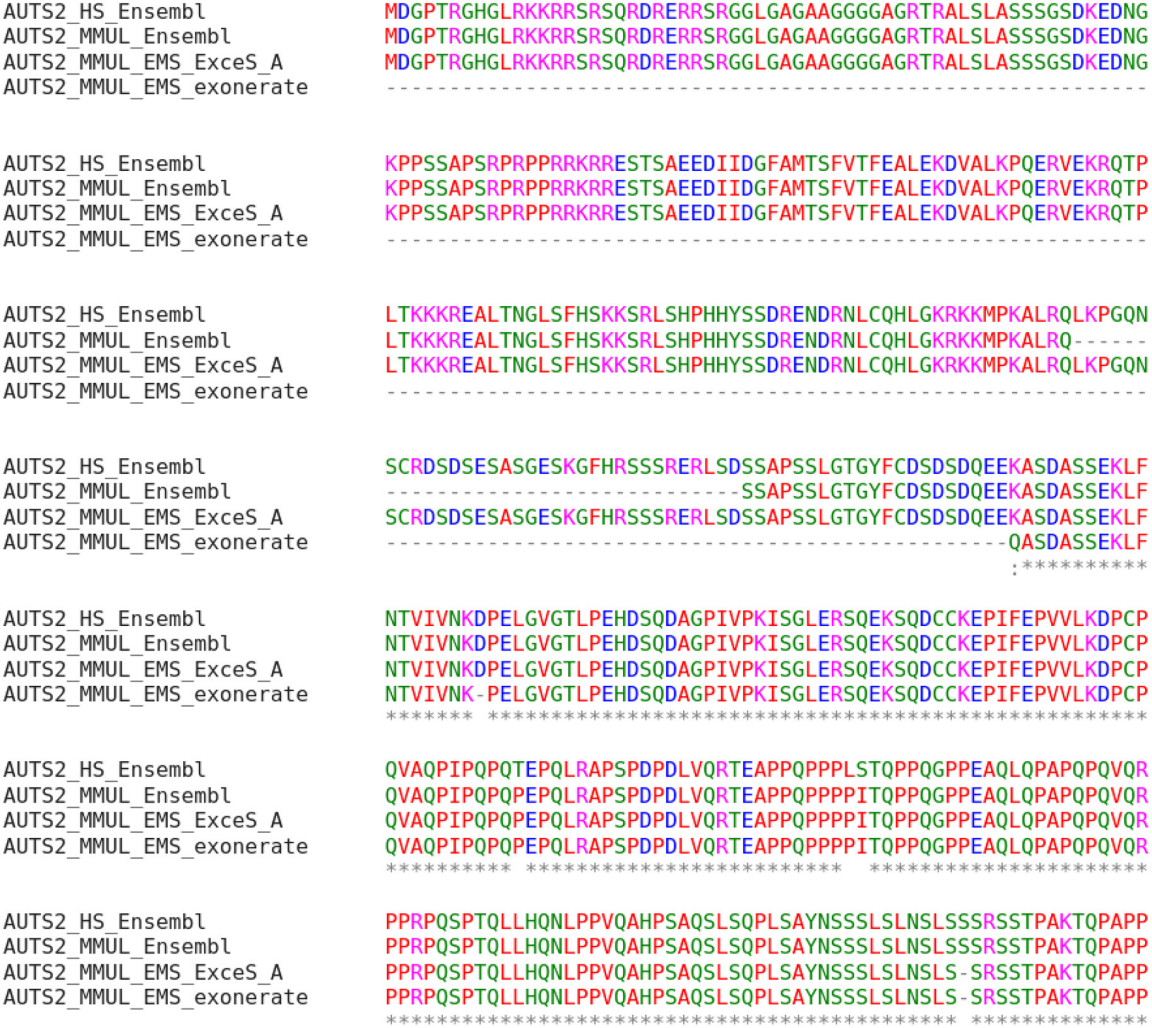
Reconstruction of AUTS2 in *Macaca mulatta* (MMUL) using the human (HS) protein. The ExonMatchSolver pipeline (EMS) is used with ExceS-A and the combination of Exonerate and ProSplign as spliced aligners. The Ensembl annotation for the macaque AUTS2 is shown for comparison. ExceS-A identifies perfectly preserved orthologous sequences, while Exonerate fails to identify the 5′ part of the molecule due to the long intron. The Ensembl annotation also misses one exon.

The AUTS2 gene is an example of a gene with extreme long ( >300,000 nt) introns. It has been implicated in neurodevelopment and is a candidate gene for numerous neurological disorders, see e.g. [18]. Human AUTS2 comprises 19 exons, while the Ensembl database AUTS2 has 18 exons in the *Macaca mulatta* (macaque) genome. Using the human AUTS2 protein as a query, ProSplign fails to retrieve a macaque homolog. Exonerate finds at least 14 exons in macaque. ExceS-A, on the other hand, annotates all 19 exons with high sequence similarity (Figure 4), indicating a complete conservation of the gene structure.

A run of the full workflow using the *Homo sapiens* AUTS2 sequence as query and 26 primate genomes target reveals a very high level of both sequence similarity and conservation of exon/intron structure among all identified orthologs. We also observe that the intron lengths are well preserved within this data set. Our ExceS-A-based workflow identified many additional exons compared to the Ensembl annotation for the corresponding species, see S3 in the Supplemental Material. The predicted AUTS2 protein sequences and HMMs of the exons are also available in the Supplemental Material (S4 and S5).

**Figure 5:**
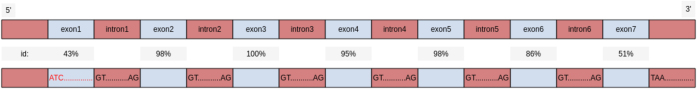
NPR5 in *Caenorhabditis japonica*. The ProSplign annotation of NPR5 in *Caenorhabditis japonica* includes exon1 missing a start codon (The first exon starts with ATC (red). The identity score for this exon is only 43% as measured by ProSplign. While missing a start codon in the first exon, this exon is missing in the ExceS-A output for this protein.

The ProSplign annotation of the NPR5 sequence in *Caenorhabditis japonica* (NPR5 sequence from *Caenorhabditis elegans* as query) includes a first exon (exon1) that lacks a canonical start codon (the identified sequence starts with ATC instead) and, in contrast to other exons, has a very low identity score compared to the query. ExceS-A therefore rather omits this exon (Figure 5).

As component of the ExonMatchSolver, ExceS-A reduces the number of initial candidate exons since it excludes weak, ambiguous predictions. In an application to the family of Neuropeptide Y/RFamide-like receptors, which contains 41 members in *C. elegans*, this simplifies the input for the paralog-to-contig assignment problem. In ExonMatchSolver, this task is solved by integer linear programming (ILP), which becomes the performance bottleneck for large inputs. In this example, the number of initial matches passed to the ILP was reduced to about one third. At the same time, we predict more paralogs and utilize more distinct contigs, resulting in the end in gene models with few exons due to the exclusion of highly diverged sequences. At the same time, the number of paralogs in the final result increased, while the number of exons decreased in the ExceS-A-based results compared to the ProSplign annotation, since exons without credible splice sites are excluded. Further details can be found in the Supplement (Table S1).

## 4 Discussion

It is still a difficult problem to reliably identify and distinguish the members of multiple paralog groups in large gene families. Often this cannot be done reliably based on pairwise genomic comparisons. Poorly assembled genomes, in which in particular genes with large introns are distributed over multiple exons, aggravate this problem. The explicit inclusion of the exon/intron structure in the comparisons can substantial improve the accuracy of orthology assignments. For large-scale applications, this requires a fast, exon-centric spliced aligner. ExceS-A provides a simple and robust solution for this purpose.

In contrast to tools such as ProSplign and Exonerate, ExceS-A focuses on high-scoring splice junctions to place its best guess for the splice sites. This is motivated by the fact that our primary application requires accurate estimates of exon boundaries, which is less of a concern if the main goal is the prediction of complete protein sequence only. For future work, it would seem natural to investigate whether even better results can be obtained by integrating a local split alignment of the sequence flanking the splice space with the scoring of the splice junction. This could be done e.g. in a dynamic programming algorithm as in segemehl [19] by using (properly scaled) combined MaxEntScan scores to modify the score of the “jump” cost similar to the split alignments. Alternatively, one could include the insertions/deletions relative to the position of splice junction in the query sequence into the scoring of splice junction.

A second difference is that conventional tools are optimized to identify global alignments and thus are prone to include also poorly scoring exons into the predicted sequence. In contrast, ExceS-A tends to exclude poor matches altogether, treating them exon losses. For our applications this behavior is desired since HMM models suffer much more from the inclusion of an erroneous, effectively random sequences than from occasional missing data.

Since ExceS-A and conventional spliced aligners such as ProSplign or Exonerate address both conceptually and technically related but distinct problems we have not attempted a quantitative benchmark. As the example of AUTS2 shows, furthermore, it is at least difficult to obtain a gold-standard set of real-life data, since available annotation tracks are likely plagued by incomplete proteins in particular for the difficult cases with long introns and short exons. The ExceS-A pipeline was developed for applications at moderate phylogenetic depths. Since it relies on blat in the initial step, we fully expect that performance will deteriorate for highly divergent sequences, i.e., as soon as blat does not reliably retrieve homologs.

## Availability

An implementation of the ExceS-A workflow and accompanying data are available at https://github.com/frarei312/ExceS-A-An-Exon-Centric-Split-Aligner.

## Supplementary Material

Supplementary Material Details
