# Supplementary material for: ExceS-A: an exon-centric split aligner
Source: J Integr Bioinform. 2022 Mar 7;19(1):20210040. doi: 10.1515/jib-2021-0040 (PMC9069663; doi:10.1515/jib-2021-0040)
Supplement: Supplementary file 1 — Supplementary Material Details [file j_jib-2021-0040_suppl.zip › Supplemental_material/S1_S2.pdf]

Supplemental Material

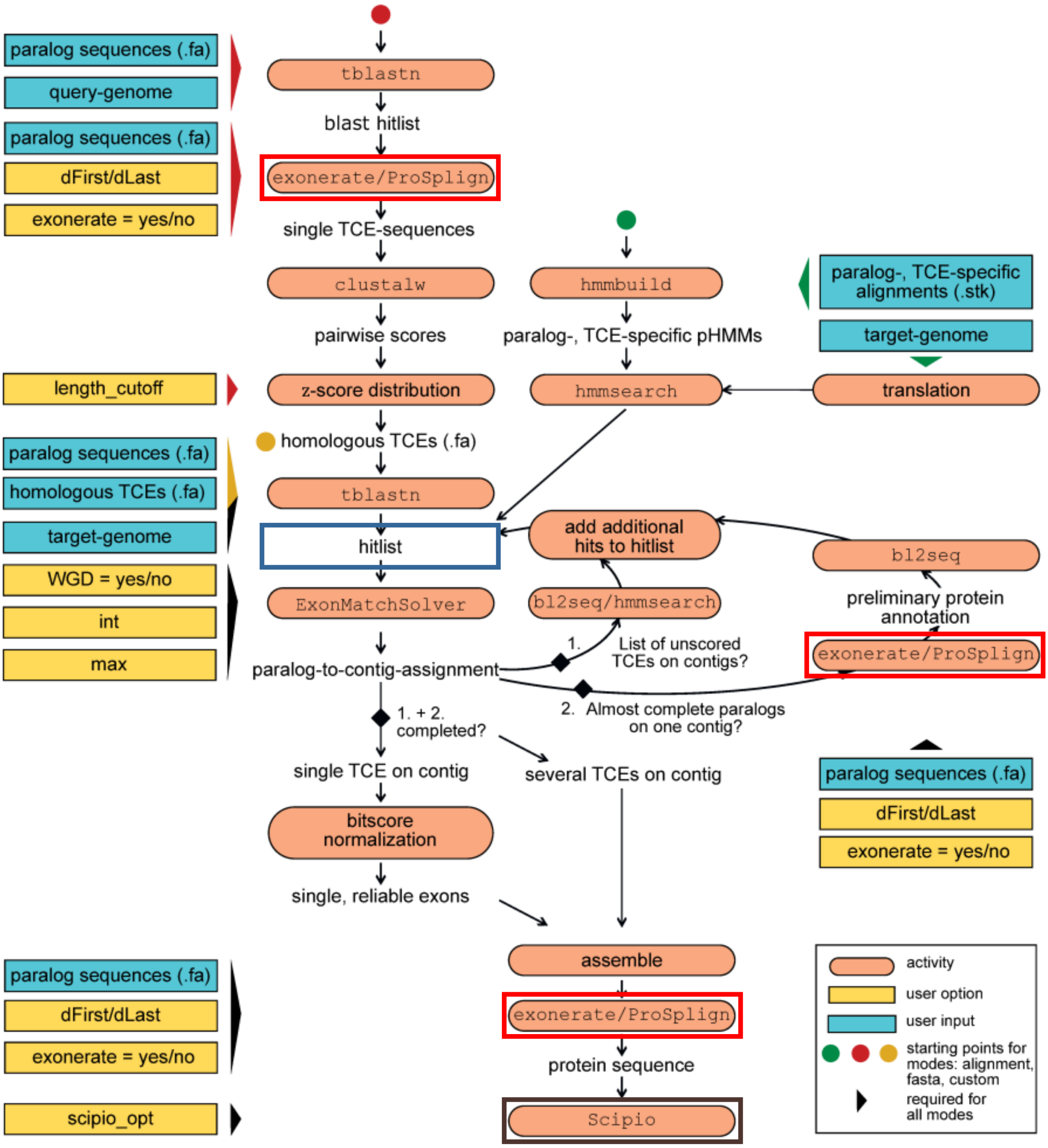

Figure S1: *ExonMatchSolver* ***pipeline including ExceS-A . Outlined in red:*** *Exonerate/ProSplign* ***is replaced by ExceS-A . Outlined in blue: hitlist will also refined by ExceS-A before ExonMatchSolver-ILP. Outlined in brown:*** *Scipio* ***is no longer required. Figure according to Indrischek et al. [1]***

	EMS pipeline with *ProSplign/Exonerate*	EMS pipeline with ExceS-A
paralog sequences in Summary.fa	19	21
entries EMS1.input	1570	559
entries EMS2.input	1570	559
entries EMS3.input	1570	763
mapped contigs EMS1.out last Solution	22	27
mapped exons EMS1.out last Solution	181	149
mapped contigs EMS3.out last Solution	22	25
mapped exons EMS3.out last Solution	181	149

Table S1: ***Comparison** $ExonMatchSolver$ **(EMS) pipeline with** $ProSplign/Exonerate$ **and ExceS-A on the example of** 17 **paralogous Neuropeptide Y/RFamide-like receptor sequences in C. japonica. The entries of** $EMS.input$ **refer to the*** **hitlist** ***indicated by a blue outline in Fig. S1.*** $ExonMatchSolver$ ***re-computes the solution of paralog-to-contig assignment problems a maximum of three times and additional hits, if any, are added to the hitlist after each run. In each iteration, the mapped contigs and exons of the ILP solution are listed in the file*** $EMS.out$***. The maximal number of solutions is determined by the user using the*** $maxParalogs$ ***parameter, which limits the number of paralog to be considered. We ran the pipeline with the same value of*** $maxParalogs = 20$ ***using either*** $Exonerate/ProSpline$ ***and ExceS-A , respectively, to handle the spliced alignments. ExceS-A retrieves more paralogs mapping to different contigs than*** $ProSplign/Exonerate$ ***but still includes fewer exons.***

References

1 Indrischek H, Wieseke N, Stadler PF, Prohaska SJ. The paralog-to-contig assignment problem: high quality gene models from fragmented assemblies. Algorithms for Molecular Biology. 2016;11(1):1–14.
